# College Lecture Fees

**Published:** 1879-08

**Authors:** 


### ARTICLE XVII.

### COLLEGE LECTURE FEES.

We see by the annual announcements of the several colleges, that all the regular medical colleges in Chicago and Cincinnati have placed the annual lecture fees at $75, instead of ranging from $40 to $55, as heretofore. This is a judicious move in the right direction, and is all the more commendable from being done, simultaneously, by all the more important schools in the West, whose trustees and faculties, had in former years allowed the influence of the substantially free medical schools attached to the State Universities of Michigan and Iowa, to depress their lecture fees to an unduly low figure.

---

PPOSPHATURIA IN ITS RELATION TO SURGICAL OPERATIONS. (*Progrès Médical.* M. Verneuil.) M. Verneuil called the attention of the Academy of Medicine to a recent thesis by Mr. Tessier, of Lyons, in which he reported certain cases of phosphaturia associated with certain ocular affections, which on that account became exceedingly troublesome. M. V., having his attention drawn to the subject by this thesis of Mr. Tessier, soon observed analagous cases. A small fibroid tumor was removed from the thigh. The operation was in itself insignificant, yet troublesome hæmorrhage followed, considerable suppuration, the cure requiring a period of six months. During this period it was ascertained that the urine contained large quantities of earthy phosphates. He also observed that in an individual, the subject of phosphaturia, or excessive discharge of phosphates, fractures would seem to be more readily effected, and the union more tardy. It appears from M. V.'s paper that phosphaturia retards reparation of wounds, and render the bones more fragile. Looking at the question more generally, M. V. concludes that the phosphaturia was not at all the *result* of the wounds or fractures, but more or less the *cause* of the fractures and the tardy union of wounds.

